# A stochastic model of oncogene expression and the relevance of this model to cancer therapy

**DOI:** 10.1186/1742-4682-3-5

**Published:** 2006-01-31

**Authors:** Francis D Alfano

**Affiliations:** 1 The Harold Leever Cancer Center, 1075 Chase Parkway, Waterbury, Connecticut, 06708, USA

## Abstract

**Background:**

Ablation of an oncogene or of the activity of the protein it encodes can result in apoptosis and/or inhibit tumor cell proliferation. Therefore, if the oncogene or set of oncogenes contributing maximally to a tumor cell's survival can be identified, such oncogene(s) are the most appropriate target(s) for maximizing tumor cell kill.

**Methods and results:**

A mathematical model is presented that describes cellular phenotypic entropy as a function of cellular proliferation and/or survival, and states of transformation and differentiation. Oncogenes become part of the cellular machinery, block apoptosis and differentiation or promote proliferation and give rise to new states of cellular transformation. Our model gives a quantitative assessment of the amount of cellular death or growth inhibition that result from the ablation of an oncogene's protein product. We review data from studies of chronic myelogenous leukemia and K562 cells to illustrate these principles.

**Conclusion:**

The model discussed in this paper has implications for oncogene-directed therapies and their use in combination with other therapeutic modalities.

## Background

For the past thirty years, cancer research has elucidated a family of genes that are integrally involved in the cancer process. These genes comprise two subsets. One subset, termed oncogenes, gives rise to proteins that modulate such processes as cell cycle progression, signaling, cellular growth and apoptosis [1-3]. The other consists of genes that can suppress tumor activity and their absence can lead to the initiation and/or progression of cancer [4]. There has been a body of work discussing the view that cancer arises from genetic instability. Evidence for genetic instability that has been cited includes the occurrence of chromosomal abnormalities, microsatellite DNAs and aberrant gene expression through hypermethylation of DNA [5-7]. Moreover, recent work using RNA microarray analysis has shown that there are key genes that are overexpressed as a result of malignant transformation, and others that are under expressed, compared to RNA transcripts in nonmalignant counterparts [8]. Many of these gene expression changes illustrated by gene microarray analysis may be secondary or even far distal to the primary changes determined by the actual oncogene or suppressor gene. In order to define the complex behavior of a tumor cell population associated with these complex gene expressions, we have chosen to define entropies of apoptosis, cellular differentiation and survival or growth inhibition. We hypothesize that a cell's phenotypic entropy is determined as a function of the survival fraction or proliferation rate of a tumor ;and also, the number of transformed and differentiated states that arise within a particular cell population. The mathematical relations that we have formulated can quantitatively determine how ablation of an oncogene's protein activity can result in apoptosis and/or a decrease in proliferation within a population of tumor cells. The goal is then to determine which oncogene or set of oncogenes contributes maximally to a tumor cell's survival; and thereby, to predict which oncogene(s) are the most appropriate target(s) for maximizing tumor cell kill.

### The model

The cellular phenotypic entropy is determined by first defining all allowable phenotypes. These phenotypes include the set of transformed states associated with aberrant gene expression, the set of differentiated states that have been defined as a result of stochastic gene expression and the distribution of cells between living and dead or growth inhibited. Therefore

f_s _Ent(phenotype) = Ent(transform/differentiation) f_s _+ Ent(cell survival)     (1)

where Ent(transform/differentiation) is the entropy of transformation and differentiation per observed population and Ent(cell survival) is the entropy of cell death or growth inhibition per total population; f_s _is the ratio of observed cells to predicted cells.

The transformed states are the phenotypes that a cell can access which provide a hyperproliferative advantage over the cell's normal counterpart. This includes phenotypes that have a better growth advantage as well as phenotypes that have antiapoptotic behaviors in environments that would normally lead to cellular apoptosis. The total number of states that a cell can access is determined by the number of end-differentiated states and by the number of new "environments" that a transformed cell can inhabit over and above its normal counterpart. Let Ω represent the number of transformed states that a cell can access as a transformed cell and let ω represent the number of states that the cell's normal counterpart can access via differentiation. We presume that ω is equal to the number of end-differentiated states in an undifferentiated cell or equal to 1 in an end-differentiated cell. We will define the entropy of the combined states of transformation and differentiation as

f_s _Ent(transform/differentiation) = c f_s _ln(ω Ω + ω).     (2)

Equation (2) is motivated by the classic definition of entropy as applied to biological systems [9]. If a system can exist in N equivalent configurations, then the entropy of that system is given by

Entropy = c ln N

where "c" is a constant of proportionality.

In equation (2), we presume that each purely transformed state will ultimately interact with each differentiated state giving rise to a new and unique transformed state. For example, an undifferentiated cell containing a transformed phenotype and differentiating into two differentiated cells will occupy one of two possible transformed phenotypes.

**Figure 1:**
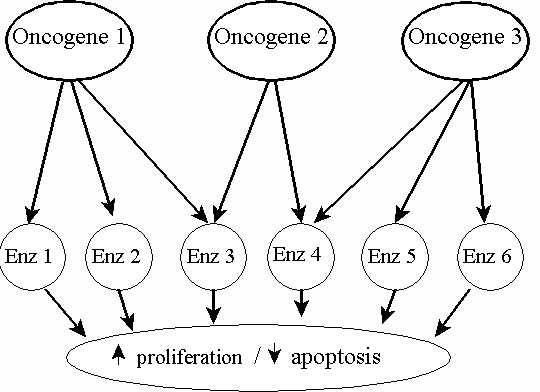
Oncogenes can affect multiple cellular pathways, which result in modulating proliferation and apoptosis. Depicted here are three oncogenes. Oncogene 2's activity overlaps with the activities of the other two. Therefore, ablation of oncogene 2's activity would not result in any measurable change in proliferation or apoptosis.

Each transformed state confers a survival advantage to the transformed cell as compared with the cell's normal counterpart. More practically, transformed cells are also defined by the set of oncogene/suppressor genes that are active within the cell and the number of different cellular mechanisms that this set of genes acts upon to change the cell's behavior with respect to growth and apoptosis. To simplify further analysis, let us consider the action of oncogenes only and disregard the action of suppressor genes. Also, we will assume that there is a direct correlation between the set of 'environments' that a transformed cell can inhabit and the unique cellular mechanism that an oncogene affects. Therefore, let us correlate each transformed state Ω with the number of oncogenes multiplied by the unique cellular mechanisms that each oncogene affects (see Fig. 1).

The term Ent(cell survival) in equation (1) can be calculated by defining cell death (or decreased proliferation) and cell viability (or enhanced viability) as two states that are independent of the number of transformed states but still nonetheless contribute to the overall phenotypic entropy. Define the total number of cells, which is determined by a suitable control, as N_c _and the measured number of observed cells as n_s_. The difference, N_c_-n_s_, in some cases would represent apoptosis or cell death, and in other cases would represent decreased proliferation measured against a suitable standard. If Ent(cell survival) is defined in a canonicalthermodynamic formalism [9], then

N_c _Ent(cell survival) = c ln(N_c_!/n_s_!(N_c_-n_s_)!).     (3)

Utilizing Stirling's approximation [10] and defining f_s _= n_s_/N_c_, Equation (3) becomes

Ent(cell survival) - c [(1-f_s_) ln(1-f_s_) +f_s _ln f_s_] for 0<f_s_<1     (4)

and

Ent(cell survival) = 0 for f_s _= 0 or 1 by continuity.

Combining equations (1), (2) and (4) gives us

f_s _[Ent(phenotype)] = - c [(1-f_s_) ln(1-f_s_) +f_s _ln f_s_] + c f_s _ln(ω Ω + ω) or

Ent(phenotype) = - c [((1-f_s_)/f_s_)ln(1-f_s_)+ ln f_s_] + c ln(Ω + 1)+ c ln(ω).     (5)

Equation (5) is the general statement of the model, which relates the phenotypic entropy to the processes of differentiation, transformation and cellular growth and/or apoptosis.

Since the entropy in equation (5) is defined along the classic definition of entropy, we can apply the second law of thermodynamics to our analysis. Consider a modulator of differentiation and/or oncogene activity that either reduces or completely eliminates the action of the oncogene or changes the number of available differentiated phenotypes. We know that Ent(phenotype) should increase or remain equal with time, and therefore this entropy, after administration of the oncogene modulator, should be greater than or equal to the entropy prior to its administration. However, if the inhibitor is removed, then the entropy after removal should return to that of the premodulator's environment, i.e.

Ent(phenotype)_premodulator _≤ Ent(phenotype)_postmodulator _≥ Ent(phenotype) _premodulator_.     (6)

The result is self-consistent only if equation (6) is considered with the equal signs. Therefore, if we take times pre and post modulator administration that most closely approximate the equality of entropies pre and post the administration of the modulator, then

{- [((1-f_s_)/f_s_) ln(1-f_s_)+ ln f_s_] + ln(Ω + 1)+ ln(ω)}_premodulator _= {- [((1-f_s_)/f_s_) ln(1-f_s_) + ln f_s_] + ln(Ω + 1)+ ln(ω)}_post_modulator_.     (7)

In Table 1, we consider multiple examples where changes of Ω and ω occur as a result of the modulator; f_s _(pre modulator) is for most of the examples taken as 1 but we consider examples of modulator given in the setting of cytotoxic drugs, which can reduce f_s _(premodulator) to less than 1. The intent of these substitutions is to calculate f_s _(post modulator) to determine the effect of the modulator in different settings. We can solve for f_s _(post modulator) in equation (7) by using the *root *function of MATHCAD version 11 [25].

**Table 1:** Tumor cell survival fraction as a function of changes in the states of transformation, differentiation, and the initial tumor survival fraction.

f_s_(Pre)	Ω(Pre)-> Ω(Post)	ω(Pre)->ω(Post)	f_s_(Post)
1.0	3->0	1->1	.50
1.0	3->1	2->2	.77
1.0	3->1	2->1	.50
1.0	3->2	3->2	.77
1.0	3->2	3->3	.92
.85	3->2	3->3	.74
.85	3->2	3->2	.57

The term f_s_(Pre) is the survival fraction of a tumor cell's population prior to the application of a targeted therapy; Ω(Pre)-> Ω(Post) represents the change in transformed states resulting from oncogene inhibition ; ω(Pre)->ω(Post) represents the change in differentiation states resulting from oncogene inhibition. The term f_s_(Post) is the survival fraction or reduced proliferation of a tumor cell population that results from oncogene inhibition and is calculated from equation (7).

### Chronic mylogenous leukemia and K562

Our intent is to study oncogenic behavior in realistic models to determine whether the principles outlined above can predict outcomes of therapy. As an example, let us consider the Bcr-Abl oncogenic protein, which is the transforming agent for chronic myelogenous leukemia (CML) [11,12]. The Bcr-Abl protein is the result of the fusion of sequences from the Abl proto-oncogene on chromosome 9 with the sequences from the proto-oncogene, Bcr, on chromosome 22. The two major forms of Bcr-Abl, p210 and p190, can each cause chronic myelogenous leukemia (CML) in humans. The Abl component of this protein encodes a nonreceptor tyrosine kinase that is constitutively active and activates a number of signal transduction pathways involved with cell proliferation and apoptosis. Bcr-Abl can inhibit apoptosis and decrease cell proliferation by its kinase action in experimental systems and myeloid cells. These mechanisms have been shown to be mediated for the most part through (1) activation of phosphatidylinositol 3-kinase (PI-3K) and (2) Jak-Stat kinases. In addition, Bcr-Abl can affect p53 and MYC in a RAS-dependent manner [12-14] and can activate Jun N-terminal kinase (JNK).

**Figure 2:**
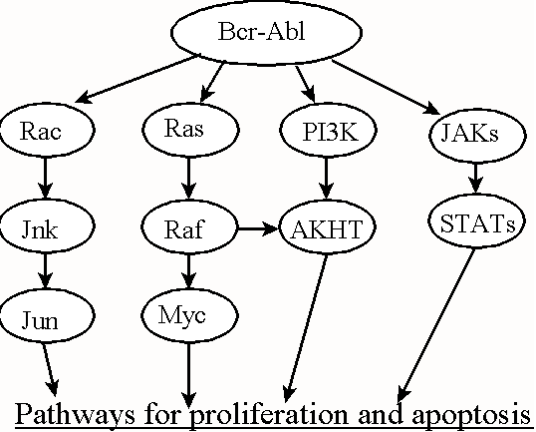
Bcr-Abl modulates up to four cellular pathways, but because of the interdependence of these pathways, we conclude that 3 pathways best represent the number of transformed states incurred by the oncogene's behavior.

In CML, Marley et al. [15,16] found that the antiproliferative effect of the Bcr-Abl inhibitor Imatinib correlated most closely with the inhibition of PI-3K within chronic myeloid leukemia progenitor cells, and also found that AG490, a Jak2 kinase inhibitor and FTI II, a farnesyltransferase inhibitor and an inhibitor of RAS activation, could also reduce the proliferation of clonogenic CML cells. This suggests that Bcr-Abl can influence at least three separate proliferation or antiapoptotic mechanisms within CML cells and effect transformation by activating three separate cellular mechanisms (see Fig. 2). There is evidence that CML is a disease of stem cells that can undergo self-renewal as well as differentiate into committed progenitor cells capable of proliferating. Laboratory evidence has shown that drugs such as interferon or Imatinib, the inhibitor of the Bcr-Abl kinase, have different antiproliferative effects on CML stem cells and committed progenitor cells [17-19]. Therefore, any analysis of CML proliferation and apoptosis needs to take into account these two distinct cellular types.

Imatinib is a tyrosine kinase inhibitor that specifically binds the ATP pocket of Bcr-Abl tyrosine kinase, inhibiting the activity of the kinase. Moreover, it is known to induce apoptosis in Bcr-Abl positive cells [15,16]. In CML cells, the predominant effect of Imatinib is not to induce apoptosis but to decrease proliferation of the committed progenitor cells and to a lesser extent the CML stem cells [17,19]. We can use equation (7) and Table 1 to calculate the effect of Imatinib on the stem cell and committed progenitor populations. The committed progenitor population is a differentiated system, and therefore ω(preImatinib) = ω(postImatinib). The term [(1-f_s_)/f_s _ln(1-f_s_) +ln f_s_]_preImatinib _is taken to be zero since f_s_(preImatinib) is taken to be nearly equal to one. Since the Bcr-Abl kinase predominantly affects three enzyme mechanisms, the Ω(preImatinib) is equal to three. We will assume the maximum effect of Imatinib and so take Ω(postImatinib) to be zero. Therefore, symbolically, we have {Ω(Pre)->Ω(Post): 3->0; ω(Pre)->ω(Post): 1->1; f_s_(Pre) = 1}. By referring to Table 1, we find f_s_(Post) to be 0.5. In CML stem cells, Imatinib has been shown to have much less of an impact on cellular proliferation. One postulated mechanism for this is the presence of an enhanced multidrug resistance protein (MDR) which extrudes the drug from the interior of the cell [20]. Therefore, Imatinib may not maximally inhibit the cellular mechanisms outlined above. We can reasonably postulate that Ω(Pre)->Ω(Post): 3->1. Furthermore, the effect of Imatinib on differentiation of the CML stem cell is not clear. Schuster et al. [21] were able to show that the block of differentiation on a murine hematopoietic progenitor line by Bcr-Abl kinase was reversed by Imatinib but Angstreich et al. [22] were unable to show any effect on the differentiation of CML progenitor stem cells by Imatinib. For our analysis of CML stem cells, we will consider a mixing of two states; i.e. {Ω(Pre)->Ω(Post): 3->1; ω(Pre)->ω(Post): 2->2; f_s_(Pre) = 1} and {Ω(Pre)->Ω(Post): 3->1; ω(Pre)->ω(Post): 2->1; f_s_(Pre) = 1} to reflect this duplicity of differentiation data. Table 1 shows that f_s _= 0.77 and 0.5 for these two situations, respectively. This establishes the range of f_s _to be between 0.5 and 0.77 for CML stem cells.

Holtz et al. [19] studied the separate effects of Imatinib on CML stem cells and committed progenitor cells and derived an index of inhibition that is appropriate for our analysis. They found a progenitor frequency that was decreased by 52 ± 5 % for committed progenitors and 43 ± 12% for primitive progenitors (stem cells). If we associate one minus the percent decrease in progenitor frequency with f_s_, then f_s _is equal to 0.48 ± 0.05 and 0.57 ± 0.12 by their data, in agreement with our theoretical predictions.

Imatinib and the chemotherapy drug cytosine-arabinoside have been shown to induce apoptosis and erythroid differentiation in the Bcr-Abl positive cell line K562 [23]. Fang et al. [23] also demonstrated a strong influence by Imatinib on the Akt kinase system of K562 cells. Imatinib induced erythroid differentiation in 37.5 % of K562 cells. Arnaud et al. [24] also observed erythroid differentiation in response to Imatinib and furthermore observed megakaryocytic differentiation with respect to phorbol esters. Therefore, for the K562 system, one can consider a mix of the states {Ω(Pre)->Ω(Post): 3->2; ω(Pre)->ω(Post): 3->2; f_s_(Pre) = 1} and {Ω(Pre)->Ω(Post): 3->2; ω(Pre)->ω(Post): 3->3; f_s_(Pre) = 1} to represent this system. We can compute values of f_s_(Post) as 0.77 and 0.92, respectively. Fang et al. found that the percentage of nonapoptotic cells measured by an Annexin V assay was 81.7 ± 2.4% and by a morphology assay was 84.9 ± 1.6%.

When cytosine-arabinoside was added to the system, differentiation remained about the same at 38.8% but the percent of nonapoptotic cells decreased to 71.2 ± 1.8% and 65 ± 0.3% by the Annexin and morphology assays, respectively. Since cytosine-arabinoside alone induced an apoptosis of 15%, we have that f_s_(PreImatinib but in the presence of cytosine-arabiniside) was0.85. Substituting this into the above states, we have {Ω(Pre)->Ω(Post): 3->2; ω(Pre)->ω(Post):3->3; f_s_(Pre) = .85} and {Ω(Pre)->Ω(Post): 3->2; ω(Pre)->ω(Post): 3->2; f_s_(Pre) = .85}. By referring to Table 1, we find that f_s_(Post) is equal to 0.74 and 0.57, respectively. The theoretical values are within the range of the experimental data.

## Discussion

We have developed a model of cellular behavior that interprets cellular transformation, apoptosis/proliferation and differentiation as stochastic processes. The model defines the necessity of considering all these mechanisms of cellular behavior together because there is interdependence amongst them. For example, differentiation may lead to the generation of apoptosis or decreased cellular proliferation; and transformation can result in enhanced proliferation when compared to the transformed cell's normal counterpart. Others have interpreted cellular transformation as a stochastic process [9,26] and several lines of evidence have been developed to explain the underlying cause of the stochastic behavior of cancer. Genetic instability as a cause for cancer has been a recurring theme since the classic paper of Boveri [27] and is defined by most authors as the generation of altered cellular behavior because of an altered protein network secondary to the introduction of a new oncogene protein or the removal of a tumor suppressor protein. In either case, definite outcomes are thought to be predicted by either event. Furthermore, over a long enough period of time, cellular behavior can evolve within a transformed population of cells, leading to a heterogeneous set of cellular behaviors.

We have used entropy as a measure of change for transformation; not only because entropy is a linear function and often different items of interest can simply be added together, but also because this approach is supported by past analyses that have used entropy to model cancer behavior in the context of chemical carcinogenesis [9]. Furthermore, recent work on the differentiation of myeloid colony-forming cells has shown that experimental data best fit a stochastic model [28]. Because of the simplicity of the entropy function, we can collect components of cellular behavior that best fit our knowledge of the cellular phenotype; i.e. the cell's growth capacity and survival, the cell's differentiation status and the cell's transformation status. Each of these quantities can be defined within the context of an entropy function and combined to serve as an index of cellular phenotypic entropy.

We consider the cellular phenotypic entropy to remain constant during therapies that are observed to be reversible. As an example for study, we chose chronic myelogenous leukemia because this model is well defined in terms of the oncogenes involved. Imatinib induces a high rate of remissions when given in a clinical context, but when Imatinib is discontinued the disease returns to a clinical state identical to that observed before the inhibitor. This observation also has been made in vitro [29]. Such would not be the case with most chemotherapies since they are often mutagenic and exert their effect by modifying cellular DNA permanently [30].

In our analysis of Bcr-Abl kinase action in CML, we surmised that the protein is acting predominantly over three kinase systems to enhance cellular proliferation. These three systems involve pathways that have already been elucidated such as (1) the Akt kinase system, (2) the RAS dependent p53 system, and (3) the Jak-Stat kinase system [12,13]. However, these systems are not totally independent and their interdependence may serve to reduce the number of transforming states that we have designated within our mathematical computations. As such, the Jun pathway was also recognized to be affected by the BCR-ABL kinase, but it is not clear that this is strongly implicated in Bcr-Abl kinase action within the context of CML. Even if it were appropriate to consider the Jun pathway, the interdependence of these pathways may still justify equating Ω(Pre), the number of transformed states determined by the Bcr-Abl kinase, to three (see Fig. 2).

By our model, each enzyme system is correlated with a transformed state, and the more each system is affected by the inhibitor, the greater the effect the inhibitor has on reducing cellular growth and/or inducing apoptosis. Furthermore, if the inhibitor contributes to the differentiation of the transformed cell, then that also will contribute to a greater reduction of cellular proliferation and a possible increase in apoptosis. In all the instances we cited, the data supports our theoretical model.

**Figure 3:**
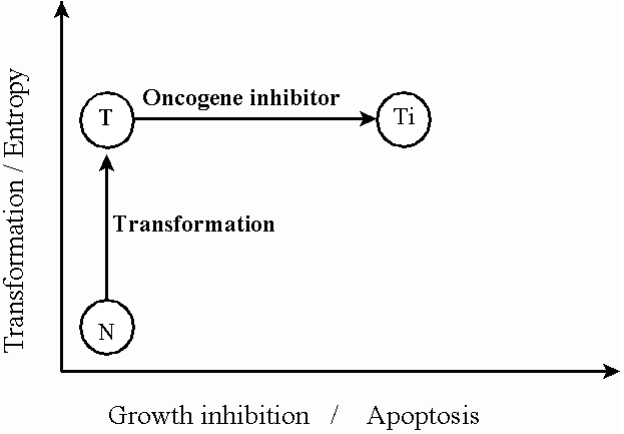
A normal cell, N, is transformed thereby increasing its phenotypic entropy. When the oncogene inhibitor is applied to the transformed cell, T, the cell maintains constant phenotypic entropy and therefore does not return to its normal state. As a result of the loss of the oncogene's protective actions, the transformed cell, Ti, is less adapted to its environment and undergoes either growth inhibition or apoptosis.

Our model does not address the issue of how a normal cell with low phenotypic entropy becomes transformed to a cell with higher entropy. Even if Imatinib fully ablates the activity of the Bcr-Abl kinase, the cell remains transformed and the phenotypic entropy does not change. Therefore, transformation by our model is considered independent of oncogene expression (see Figure 3). How can this be? The answer lies in the fact that the signature of transformation is not in the expression of the oncogene protein but in the alteration in the DNA by the oncogene. In the case of Bcr-Abl, it is the observed 9–22 chromosome translocation that affects the behavior of other normal cellular proteins [31]. Such a conclusion is supported by the observations of Keating et al., who observed variable expression of Bcr-Abl transcripts in early CML progenitor cells that exhibited the chromosome translocation [32].

More studies will be needed to determine whether this is a specific feature of Bcr-Abl positive disease or a manifestation of a more general principle of cellular transformation and cancer. Namely, does an oncogene act in a similar fashion within a set of oncogenes as it does when it acts alone? If so, then it would be important to know how to measure an oncogene's action so that one could target the specific oncogene with the greatest impact on a tumor cell's survival. Within the context of our model, we can provide a recipe for calculating the extent to which inhibiting an oncogene's action can reduce tumor cell survival by answering the following questions:

(1) How many proliferation/apoptosis mechanisms are active in the tumor cell's normal counterpart? (In CML, we argued for three mechanisms.)

(2) What oncogenes inhibit which mechanisms? The answer would most likely be specific to the oncogenes that are active within the tumor cell.

(3) How many end-differentiated states apply to the tumor's specific environment, and does the oncogene inhibitor change the number of differentiated states expressed after oncogene ablation, and does the inhibitor completely ablate the oncogene's action with respect to all proliferation/apoptosis mechanisms? And finally,

(4) What is the initial survival fraction of the tumor cell's population prior to oncogene inhibition? The initial survival may vary depending upon other therapies applied such as radiation and/or chemotherapy. Table (1) yields theoretical calculations of survival fractions as a function of changes of oncogene expression, differentiation and the initial survival fraction before a targeted therapy is applied, demonstrating the synergy between oncogene specific therapies and other modalities such as chemotherapy.

## Competing interests

The author(s) declare that they have no competing interests.
